# Tetra­aqua­(2-hy­droxy­acetato-κ^2^ *O* ^1^,*O* ^2^)magnesium nitrate

**DOI:** 10.1107/S1600536811006611

**Published:** 2011-02-26

**Authors:** Wen-Jing Liu, Zhi-Qiang Wei, Shan-Tang Yue

**Affiliations:** a School of Chemistry and Environment, South China Normal University, Guangzhou 510006, People’s Republic of China

## Abstract

In the title complex, [Mg(C_2_H_3_O_3_)(H_2_O)_4_]NO_3_, the Mg^II^ cation is hexa­coordinated by four O atoms from water mol­ecules and two O atoms from a 2-hy­droxy­acetate ligand in a distorted octa­hedral coordination geometry. The structure exhibits a three-dimensional supra­molecular network, which is stabilized by nine different O—H⋯O hydrogen bonds.

## Related literature

For related magnesium complexes, see: Erxleben & Schumacher (2001 ▶).
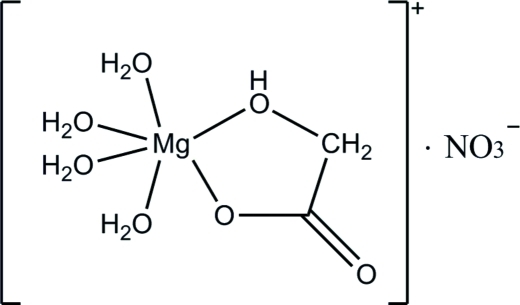


## Experimental

### 

#### Crystal data

[Mg(C_2_H_3_O_3_)(H_2_O)_4_]NO_3_
*M* *_r_* = 233.43
Monoclinic, 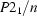

*a* = 5.777 (2) Å
*b* = 7.171 (3) Å
*c* = 23.045 (8) Å
β = 92.839 (4)°
*V* = 953.5 (6) Å^3^
*Z* = 4
Mo *K*α radiation
μ = 0.23 mm^−1^
*T* = 298 K
0.20 × 0.18 × 0.18 mm

#### Data collection

Bruker SMART CCD area-detector diffractometer
Absorption correction: multi-scan (*SADABS*; Bruker, 2001 ▶) *T*_min_ = 0.956, *T*_max_ = 0.960
4632 measured reflections
1713 independent reflections
1424 reflections with *I* > 2σ(*I*)
*R*_int_ = 0.027

#### Refinement

*R*[*F* ^2^ > 2σ(*F* ^2^)] = 0.044
*wR*(*F* ^2^) = 0.115
*S* = 1.06
1713 reflections
141 parameters
H atoms treated by a mixture of independent and constrained refinement
Δρ_max_ = 0.29 e Å^−3^
Δρ_min_ = −0.18 e Å^−3^

### 

Data collection: *SMART* (Bruker, 2001 ▶); cell refinement: *SAINT* (Bruker, 2001 ▶); data reduction: *SAINT*; program(s) used to solve structure: *SHELXTL* (Sheldrick, 2008 ▶); program(s) used to refine structure: *SHELXTL*; molecular graphics: *SHELXTL*; software used to prepare material for publication: *SHELXTL*.

## Supplementary Material

Crystal structure: contains datablocks global, I. DOI: 10.1107/S1600536811006611/go2005sup1.cif

Structure factors: contains datablocks I. DOI: 10.1107/S1600536811006611/go2005Isup2.hkl

Additional supplementary materials:  crystallographic information; 3D view; checkCIF report

## Figures and Tables

**Table 1:** Selected bond lengths (Å)

Mg1—O3*W*	2.021 (2)
Mg1—O1*W*	2.033 (2)
Mg1—O2	2.0467 (19)
Mg1—O4*W*	2.052 (2)
Mg1—O2*W*	2.058 (2)
Mg1—O1	2.069 (2)

**Table 2:** Hydrogen-bond geometry (Å, °)

*D*—H⋯*A*	*D*—H	H⋯*A*	*D*⋯*A*	*D*—H⋯*A*
O3*W*—H3*W*1⋯O2^i^	0.85	1.88	2.719 (3)	167
O3*W*—H3*W*2⋯O6^ii^	0.85	2.05	2.860 (3)	160
O1—H3⋯O3^i^	0.88 (3)	1.76 (3)	2.638 (3)	172 (3)
O1*W*—H1*W*2⋯O4^iii^	0.85	1.90	2.747 (3)	173
O1*W*—H1*W*1⋯O6^iv^	0.85	2.06	2.905 (3)	174
O4*W*—H4*W*1⋯O3^v^	0.85	1.85	2.687 (3)	166
O4*W*—H4*W*2⋯O4^vi^	0.85	2.31	3.014 (3)	141
O2*W*—H2*W*1⋯O4	0.85	2.02	2.860 (3)	169
O2*W*—H2*W*2⋯O5^vii^	0.85	2.00	2.826 (3)	166

Symmetry codes: (i) 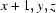; (ii) 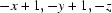; (iii) 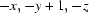; (iv) 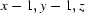; (v) 
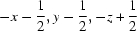
; (vi) 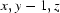; (vii) 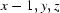.

**Fig. 1.**
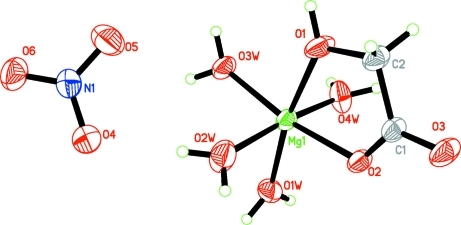
Displacement ellipsoid plot (40% probability level) of the title compound.

**Fig. 2.**
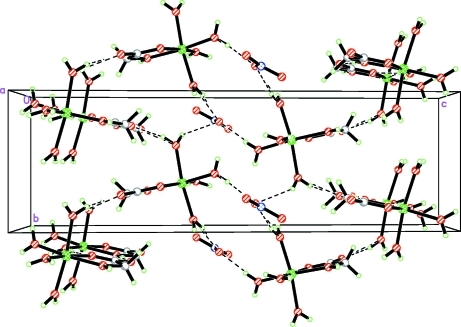
The packing diagram of the title compound.

## supplementary crystallographic information

### Comment

The compound crystallizes in the monoclinic system, space group *P*2_1_/n, an ORTEP view is shown in Fig. 1. The Mg^II^ ion is hexa-coordinated by four oxygen atoms from water and two oxygen atoms from 2-hydroxyacetato ions. The Mg—O distances are in the range of 2.021 (2)—2.069 (2) Å. The O—Mg—O bond angles fall in the range of 76.73 (8)—171.89 (10) °. The C—O distances of HOCH_2_COO^-^ are within the range of 1.247 (3) Å to 1.413 (3) Å. This molecular complex exhibits a 3D structure via O—H···O hydrogen bonding interactions (Fig. 2).

### Experimental

A mixture of 2-hydroxyacetic acid (0.038 g, 0.5 mmol), Mg(NO_3_)_2_.6H_2_O (0.064 g, 0.25 mmol) and H_2_O (7 mL) was heated to 180 °C for 72 h in a 15 ml Teflon-lined stainless-steel autoclave and then cooled to room temperature at a rate of 5 °C/h. Colorless block crystals were collected and dried in air in *ca.* 48% yield based on Mg.

### Refinement

H atoms were positioned in calculated positions, with C—H = 0.93 (aromatic) and 0.96 Å (ethanol), and refined in riding mode with *U*_iso_(H) = 1.5 *U*_eq_(C) for ethanol and 1.2 *U*_eq_(C) for the others. Water H atoms were restrained, with O—H = 0.85 (1)Å and H···H = 1.29 (1) Å.

### Crystal data

**Table:**

[Mg(C_2_H_3_O_3_)(H_2_O)_4_]NO_3_	*F*(000) = 488
*M**_r_* = 233.43	*D*_x_ = 1.626 Mg m^−^^3^*D*_m_ = 1.626 Mg m^−^^3^*D*_m_ measured by not measured
Monoclinic, *P*2_1_/*n*	Mo *K*α radiation, λ = 0.71073 Å
Hall symbol: -P 2yn	Cell parameters from 1638 reflections
*a* = 5.777 (2) Å	θ = 2.8–26.0°
*b* = 7.171 (3) Å	µ = 0.23 mm^−^^1^
*c* = 23.045 (8) Å	*T* = 298 K
β = 92.839 (4)°	Block, colorless
*V* = 953.5 (6) Å^3^	0.20 × 0.18 × 0.18 mm
*Z* = 4	

### Data collection

**Table:**

Bruker SMART CCD area-detector diffractometer	1713 independent reflections
Radiation source: fine-focus sealed tube	1424 reflections with *I* > 2σ(*I*)
graphite	*R*_int_ = 0.027
φ and ω scans	θ_max_ = 25.2°, θ_min_ = 1.8°
Absorption correction: multi-scan (*SADABS*; Bruker, 2001)	*h* = −6→6
*T*_min_ = 0.956, *T*_max_ = 0.960	*k* = −8→6
4632 measured reflections	*l* = −27→27

### Refinement

**Table:**

Refinement on *F*^2^	Primary atom site location: structure-invariant direct methods
Least-squares matrix: full	Secondary atom site location: difference Fourier map
*R*[*F*^2^ > 2σ(*F*^2^)] = 0.044	Hydrogen site location: inferred from neighbouring sites
*wR*(*F*^2^) = 0.115	H atoms treated by a mixture of independent and constrained refinement
*S* = 1.06	*w* = 1/[σ^2^(*F*_o_^2^) + (0.0488*P*)^2^ + 0.7836*P*] where *P* = (*F*_o_^2^ + 2*F*_c_^2^)/3
1713 reflections	(Δ/σ)_max_ < 0.001
141 parameters	Δρ_max_ = 0.29 e Å^−^^3^
0 restraints	Δρ_min_ = −0.18 e Å^−^^3^

### Special details

**Table:**

Geometry. All esds (except the esd in the dihedral angle between two l.s. planes) are estimated using the full covariance matrix. The cell esds are taken into account individually in the estimation of esds in distances, angles and torsion angles; correlations between esds in cell parameters are only used when they are defined by crystal symmetry. An approximate (isotropic) treatment of cell esds is used for estimating esds involving l.s. planes.
Refinement. Refinement of F^2^ against ALL reflections. The weighted R-factor wR and goodness of fit S are based on F^2^, conventional R-factors R are based on F, with F set to zero for negative F^2^. The threshold expression of F^2^ > 2sigma(F^2^) is used only for calculating R-factors(gt) etc. and is not relevant to the choice of reflections for refinement. R-factors based on F^2^ are statistically about twice as large as those based on F, and R- factors based on ALL data will be even larger.

### Fractional atomic coordinates and isotropic or equivalent isotropic displacement parameters (Å^2^)

**Table:**

	*x*	*y*	*z*	*U*_iso_*/*U*_eq_	
Mg1	0.03285 (14)	0.16089 (12)	0.12732 (3)	0.0330 (3)	
O2	−0.2625 (3)	0.1820 (3)	0.17242 (7)	0.0381 (5)	
O3W	0.3575 (3)	0.1539 (3)	0.09850 (8)	0.0480 (5)	
H3W1	0.4864	0.1697	0.1172	0.080 (13)*	
H3W2	0.3969	0.1891	0.0652	0.054 (9)*	
O1	0.1570 (3)	0.2201 (4)	0.21105 (8)	0.0507 (6)	
O1W	−0.1370 (3)	0.0888 (3)	0.05123 (8)	0.0431 (5)	
H1W2	−0.1878	0.1479	0.0213	0.065*	
H1W1	−0.1967	−0.0171	0.0433	0.065*	
O4W	0.0493 (4)	−0.1211 (3)	0.14223 (9)	0.0520 (6)	
H4W1	0.0047	−0.1825	0.1712	0.078*	
H4W2	0.1424	−0.2007	0.1291	0.078*	
O2W	−0.0052 (4)	0.4340 (3)	0.10109 (10)	0.0557 (6)	
H2W1	0.0818	0.4998	0.0805	0.083*	
H2W2	−0.1280	0.4991	0.0973	0.083*	
N1	0.4889 (4)	0.6773 (3)	0.05397 (10)	0.0436 (6)	
O4	0.2764 (4)	0.6940 (3)	0.04229 (10)	0.0606 (6)	
O6	0.6310 (4)	0.7402 (3)	0.02097 (9)	0.0599 (6)	
O5	0.5496 (4)	0.5979 (5)	0.09917 (11)	0.0840 (9)	
C2	−0.0077 (5)	0.2379 (5)	0.25407 (12)	0.0396 (6)	
C1	−0.2471 (4)	0.2188 (4)	0.22541 (11)	0.0337 (6)	
O3	−0.4157 (3)	0.2385 (3)	0.25663 (8)	0.0503 (6)	
H1	0.004 (5)	0.352 (5)	0.2734 (14)	0.052 (9)*	
H3	0.294 (6)	0.232 (4)	0.2291 (13)	0.048 (8)*	
H2	0.019 (5)	0.144 (5)	0.2852 (14)	0.055 (9)*	

### Atomic displacement parameters (Å^2^)

**Table:**

	*U*^11^	*U*^22^	*U*^33^	*U*^12^	*U*^13^	*U*^23^
Mg1	0.0246 (4)	0.0441 (5)	0.0305 (4)	−0.0018 (4)	0.0053 (3)	−0.0009 (4)
O2	0.0242 (9)	0.0586 (12)	0.0315 (9)	−0.0037 (8)	0.0030 (7)	−0.0057 (8)
O3W	0.0260 (10)	0.0799 (15)	0.0387 (11)	−0.0066 (9)	0.0068 (8)	0.0017 (10)
O1	0.0214 (10)	0.0941 (17)	0.0368 (10)	−0.0028 (10)	0.0024 (8)	−0.0129 (10)
O1W	0.0464 (11)	0.0480 (11)	0.0342 (10)	−0.0048 (9)	−0.0040 (8)	0.0008 (8)
O4W	0.0674 (14)	0.0443 (12)	0.0464 (11)	0.0072 (10)	0.0232 (10)	0.0089 (9)
O2W	0.0463 (12)	0.0447 (12)	0.0769 (15)	−0.0032 (10)	0.0127 (11)	0.0100 (11)
N1	0.0465 (15)	0.0436 (14)	0.0411 (13)	−0.0027 (11)	0.0062 (11)	0.0033 (10)
O4	0.0420 (13)	0.0704 (16)	0.0692 (15)	−0.0009 (11)	0.0007 (10)	0.0225 (12)
O6	0.0554 (13)	0.0775 (16)	0.0478 (12)	−0.0177 (12)	0.0120 (10)	0.0040 (11)
O5	0.0578 (16)	0.131 (2)	0.0627 (16)	0.0079 (16)	0.0018 (12)	0.0440 (16)
C2	0.0284 (14)	0.0565 (18)	0.0343 (14)	−0.0019 (13)	0.0048 (11)	−0.0107 (14)
C1	0.0268 (13)	0.0393 (14)	0.0353 (13)	−0.0008 (11)	0.0052 (10)	−0.0053 (11)
O3	0.0283 (10)	0.0824 (16)	0.0408 (10)	−0.0016 (10)	0.0083 (8)	−0.0188 (11)

### Geometric parameters (Å, °)

**Table:**

Mg1—O3W	2.021 (2)	O1W—H1W1	0.8499
Mg1—O1W	2.033 (2)	O4W—H4W1	0.8500
Mg1—O2	2.0467 (19)	O4W—H4W2	0.8499
Mg1—O4W	2.052 (2)	O2W—H2W1	0.8499
Mg1—O2W	2.058 (2)	O2W—H2W2	0.8500
Mg1—O1	2.069 (2)	N1—O5	1.223 (3)
O2—C1	1.248 (3)	N1—O6	1.232 (3)
O3W—H3W1	0.8498	N1—O4	1.250 (3)
O3W—H3W2	0.8498	C2—C1	1.509 (4)
O1—C2	1.413 (3)	C2—H1	0.94 (3)
O1—H3	0.88 (3)	C2—H2	0.99 (3)
O1W—H1W2	0.8499	C1—O3	1.247 (3)
			
O3W—Mg1—O1W	97.25 (8)	Mg1—O1W—H1W2	134.9
O3W—Mg1—O2	168.28 (8)	Mg1—O1W—H1W1	125.8
O1W—Mg1—O2	94.47 (8)	H1W2—O1W—H1W1	98.7
O3W—Mg1—O4W	89.68 (9)	Mg1—O4W—H4W1	129.0
O1W—Mg1—O4W	84.85 (9)	Mg1—O4W—H4W2	128.8
O2—Mg1—O4W	91.19 (8)	H4W1—O4W—H4W2	98.7
O3W—Mg1—O2W	90.83 (9)	Mg1—O2W—H2W1	129.4
O1W—Mg1—O2W	87.06 (9)	Mg1—O2W—H2W2	129.0
O2—Mg1—O2W	89.95 (9)	H2W1—O2W—H2W2	98.7
O4W—Mg1—O2W	171.89 (10)	O5—N1—O6	121.7 (3)
O3W—Mg1—O1	91.56 (8)	O5—N1—O4	117.7 (2)
O1W—Mg1—O1	170.61 (8)	O6—N1—O4	120.6 (2)
O2—Mg1—O1	76.73 (8)	O1—C2—C1	108.6 (2)
O4W—Mg1—O1	92.00 (10)	O1—C2—H1	112 (2)
O2W—Mg1—O1	96.07 (10)	C1—C2—H1	109.3 (19)
C1—O2—Mg1	119.44 (16)	O1—C2—H2	111.1 (19)
Mg1—O3W—H3W1	129.3	C1—C2—H2	111.3 (19)
Mg1—O3W—H3W2	125.7	H1—C2—H2	104 (3)
H3W1—O3W—H3W2	98.8	O3—C1—O2	124.6 (2)
C2—O1—Mg1	117.30 (16)	O3—C1—C2	117.5 (2)
C2—O1—H3	106 (2)	O2—C1—C2	117.8 (2)
Mg1—O1—H3	136 (2)		

### Hydrogen-bond geometry (Å, °)

**Table:**

*D*—H···*A*	*D*—H	H···*A*	*D*···*A*	*D*—H···*A*
O3W—H3W1···O2^i^	0.85	1.88	2.719 (3)	167
O3W—H3W2···O6^ii^	0.85	2.05	2.860 (3)	160
O1—H3···O3^i^	0.88 (3)	1.76 (3)	2.638 (3)	172 (3)
O1W—H1W2···O4^iii^	0.85	1.90	2.747 (3)	173
O1W—H1W1···O6^iv^	0.85	2.06	2.905 (3)	174
O4W—H4W1···O3^v^	0.85	1.85	2.687 (3)	166
O4W—H4W2···O4^vi^	0.85	2.31	3.014 (3)	141
O2W—H2W1···O4	0.85	2.02	2.860 (3)	169
O2W—H2W2···O5^vii^	0.85	2.00	2.826 (3)	166

Symmetry codes: (i) *x*+1, *y*, *z*; (ii) −*x*+1, −*y*+1, −*z*; (iii) −*x*, −*y*+1, −*z*; (iv) *x*−1, *y*−1, *z*; (v) −*x*−1/2, *y*−1/2, −*z*+1/2; (vi) *x*, *y*−1, *z*; (vii) *x*−1, *y*, *z*.
